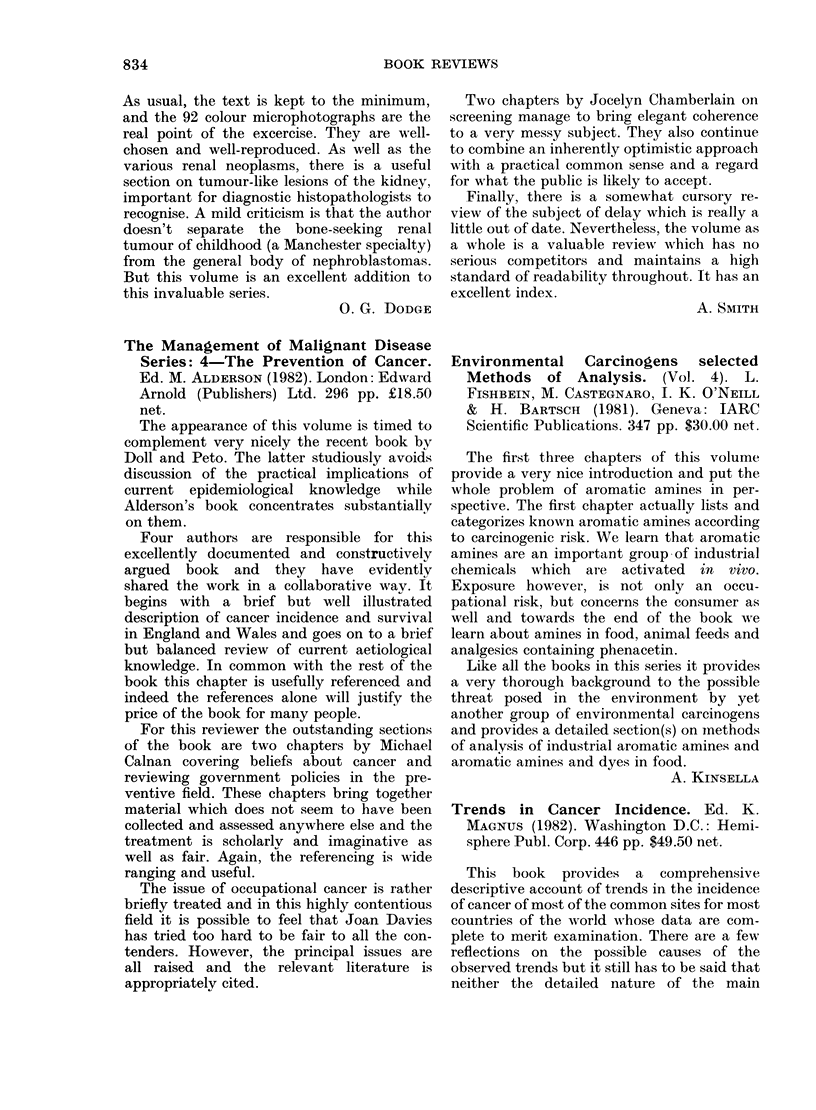# The Management of Malignant Disease Series: 4—The Prevention of Cancer

**Published:** 1982-11

**Authors:** A. Smith


**The Management of Malignant Disease Series: 4—The Prevention of Cancer.** Ed. M. ALDERSON (1982). London: Edward Arnold (Publishers) Ltd. 296 pp. £18.50 net.

The appearance of this volume is timed to complement very nicely the recent book by Doll and Peto. The latter studiously avoids discussion of the practical implications of current epidemiological knowledge while Alderson's book concentrates substantially on them.

Four authors are responsible for this excellently documented and constructively argued book and they have evidently shared the work in a collaborative way. It begins with a brief but well illustrated description of cancer incidence and survival in England and Wales and goes on to a brief but balanced review of current aetiological knowledge. In common with the rest of the book this chapter is usefully referenced and indeed the references alone will justify the price of the book for many people.

For this reviewer the outstanding sections of the book are two chapters by Michael Calnan covering beliefs about cancer and reviewing government policies in the preventive field. These chapters bring together material which does not seem to have been collected and assessed anywhere else and the treatment is scholarly and imaginative as well as fair. Again, the referencing is wide ranging and useful.

The issue of occupational cancer is rather briefly treated and in this highly contentious field it is possible to feel that Joan Davies has tried too hard to be fair to all the contenders. However, the principal issues are all raised and the relevant literature is appropriately cited.

Two chapters by Jocelyn Chamberlain on screening manage to bring elegant coherence to a very messy subject. They also continue to combine an inherently optimistic approach with a practical common sense and a regard for what the public is likely to accept.

Finally, there is a somewhat cursory review of the subject of delay which is really a little out of date. Nevertheless, the volume as a whole is a valuable review which has no serious competitors and maintains a high standard of readability throughout. It has an excellent index.

A. SMITH